# Supplementary figures and images for: Association between Cardiovascular Health and Incident Atrial Fibrillation in the General Japanese Population Aged ≥40 Years
Source: Nutrients. 2021 Sep 15;13(9):3201. doi: 10.3390/nu13093201 (PMC8467180; doi:10.3390/nu13093201)

## Slide 1
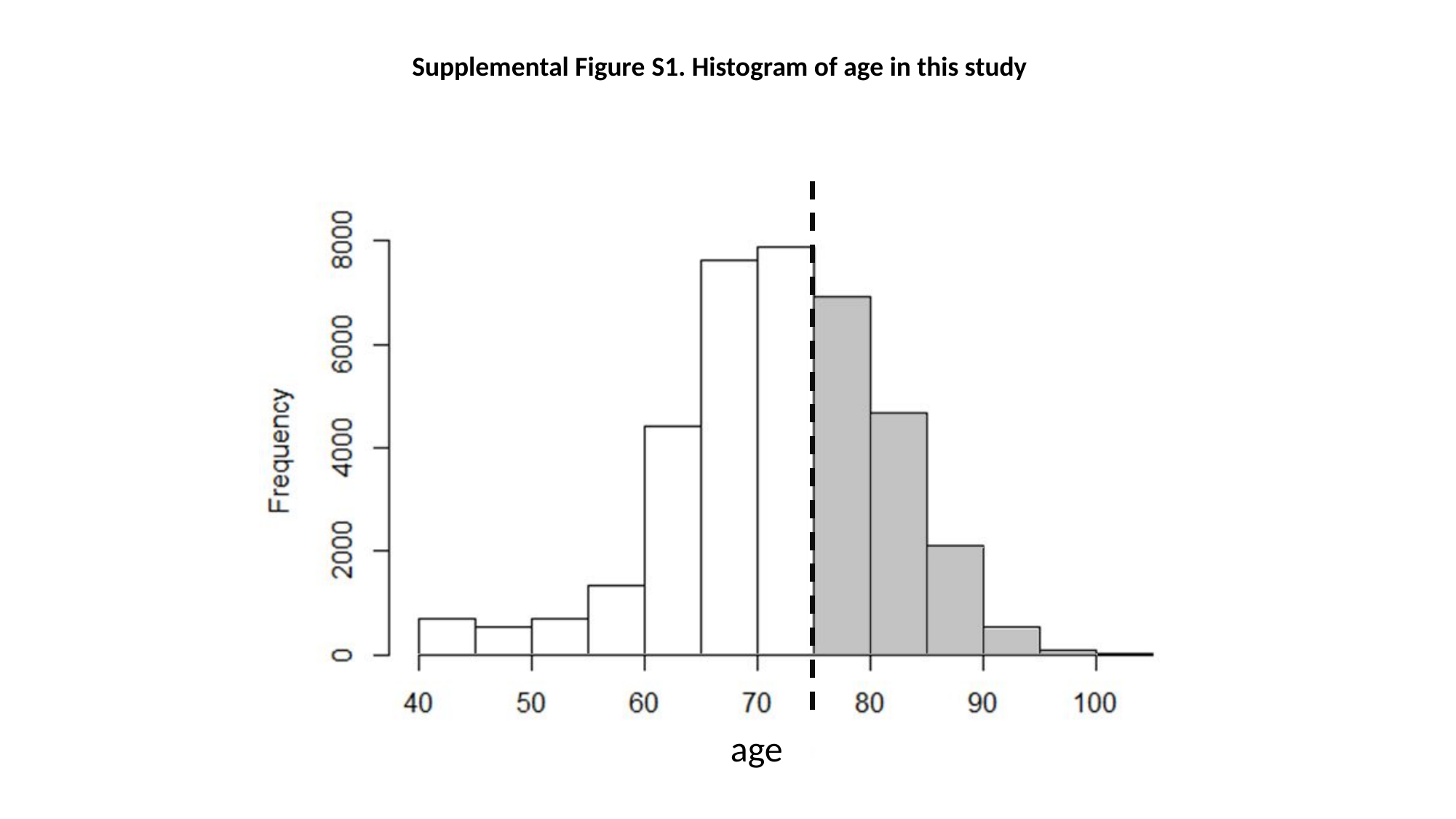

Supplemental Figure S1. Histogram of age in this study
age

Supplement: Supplementary file 1 [file nutrients-13-03201-s001.zip › nutrients-1341040-supplementary.pptx]
